# Hematopoietic Stem Cell Transplantation in Thalassemia and Sickle Cell Disease. Unicenter Experience in a Multi-Ethnic Population.

**DOI:** 10.4084/MJHID.2009.027

**Published:** 2009-12-26

**Authors:** Marco Marziali, Antonella Isgrò, Javid Gaziev, Guido Lucarelli

**Affiliations:** International Center for Transplantation in Thalassemia and Sickle Cell Anemia, Mediterranean Institute of Hematology, Rome- Italy

## Abstract

Hematopoietic stem cell transplantation (HSCT) still remains the only definitive cure currently available for patients with thalassemia and sickle cell anemia. Results of transplant in thalassemia and in sickle cell anemia have steadily improved over the last two decades due to improvements in preventive strategies, and effective control of transplant-related complications. From 2004 through 2009, 145 consecutive patients with thalassemia and sickle cell anemia, ethnically heterogeneous from Mediterranean and Middle East countries, were given HSCT in the International Center for Transplantation in Thalassemia and Sickle Cella Anemia in Rome. This experience is characterized by two peculiarities: patients were ethnically very heterogeneous and the vast majority of these patients were not regularly transfesed/chelated and therefore were highly sensitized due to RBC transfusions without leukodepletion filters. Consequently, they could have a high risk of graft rejection as a result of sensitization to HLA antigens. The Rome experience of SCT in patients with thalassemia and sickle cell anemia confirmed the results obtained in Pesaro, and most importantly showed the reproducibility of these results in other centers.

## Thalassemia:

The pathophysiology of β-thalassemia relates to a quantifiable deficiency of functional β-globin, which leads to an imbalanced globin chain production and an excess of α globin chains^1^,^2^. The latter are not able to form viable tetramers and instead precipitate in the red cell precursors forming inclusion bodies that cause mechanical damage and the premature destruction of red cell precursors in the bone marrow leading to ineffective erythropoiesis. The red cells that survive to reach the peripheral circulation are prematurely destroyed in the spleen, which becomes enlarged, eventually leading to hypersplenism. Thus, anemia in β thalassemia results from a combination of ineffective erythropoiesis, peripheral hemolysis and an overall reduction in hemoglobin synthesis leading to intense proliferation and expansion of the bone marrow with the resulting skeletal deformities.

Currently recommended non-transplant therapy is available in most countries and consists of transfusions to maintain hemoglobin levels between 9 and 10g/dL together with chelation therapy aimed at preventing iron accumulation as a consequence of the transfusion therapy. Future treatment of the thalassemic patient may be easier and improved if orally administered iron chelators become available. Two potentially useful oral chelators (Deferasirox and Deferiprone) are now available, but their efficacy compared to desferrioxamine in the long-term has not yet been determined.

## Bone Marrow Transplantation in β-Thalassemia:

The purpose of allogeneic HCT for hemoglobinopathies is to correct the basic genetic defect by repleting genes essential for normal hematopoiesis through allogeneic stem cells as vectors following conditioning to overcome the immunological barrier.

Therefore allogeneic HCT in these diseases could be considered as allogeneic stem cell gene therapy. It is a form of gene therapy that uses allogeneic stem cells as vectors for genes essential for normal hematopoiesis. Eventually, the vector may well be autologous stem cells transformed by the insertion of normal genes but there is no indication that this approach will be a clinical option in the foreseeable future.

Preparatory regimens for HCT of patients with diseases other than aplastic anemia must achieve two objectives. One is elimination of the *hemopoietic* marrow and the other is to *create* a tolerant environment that will *allow the transplanted marrow* to survive and thrive. Total body irradiation (TBI) can accomplish both these objectives, but there are many reasons to avoid the use of this marrow-ablative modality. These include the known growth-retarding effects of TBI in young children and the increased risk of secondary malignancies, which has been reported in patients treated for leukemia^3^, lymphoma and aplastic anemia^4^,^5^. These hazards are particularly objectionable in very young patients with potential for a long life span. The risk of these toxicities has not yet been fully explored for cytotoxic regimens that do not involve TBI. There is a considerable body of experience with the use of busulfan (BU) and its derivatives in ablating marrow in patients undergoing HCT for the treatment of non-malignant conditions such as the Wiskott-Aldrich syndrome^6^,^7^ and inborn errors of metabolism^8^. Cyclophosphamide (CY) is an agent that is well established as providing immunosuppression adequate for allogeneic engraftment of patients with aplastic anemia^9^,^10^. Experience in the use of chemotherapy-only transplant regimens for the treatment of malignancy^11^–^16^ has been pivotal in developing regimens appropriate for the treatment of thalassemia by transplantation.

BU is an alkylating agent with exquisite specificity for the most primitive precursors of the myeloid-erythroid axis. Clinical experience with the use of BU in very high doses was delayed due to the lack of an acceptable preparation suitable for intravenous use. Santos *et al*. reported the first clinical trials of very high-dose BU in the context of BMT^13^. In these studies, patients with acute myeloid leukemia (AML) received allogeneic marrow transplants after immunosuppression with CY (200mg/kg over 4 days), and oral BU (16mg/kg over 4 days) was administered as additional antitumor therapy. Early results with this therapy were encouraging, and in successful attempts to reduce early transplant-related toxicity, Tutschka *et al*. reduced the CY dose to 120mg/kg over 2 days 17.

Further, CY has been a component of most conditioning regimens for transplanting patients with hematologic malignancies. Santos *et al*. reported on its use in high doses (200mg/kg over 4 days) as the sole antitumor agent in patients receiving allogeneic transplants for leukemia and demonstrated that it was sufficiently immunosuppressive to permit sustained allogeneic engraftment^11^. Also, CY is most commonly employed for the treatment of lymphoid malignancies and solid tumors, and it has been used as a component of combination chemotherapy for the treatment of acute leukemia. It is not considered a highly effective agent against myeloid malignancies, although single drug studies are not available in this context. The dose-limiting toxicity of CY is to the heart and not to the marrow. Mice, monkeys and humans recover hematopoiesis promptly after the highest doses of CY because CY does not eliminate HSCs. Therefore CY alone is not an appropriate conditioning modality for BMT for the treatment of thalassemia because the thalassemic marrow would recover rapidly. On the other hand, BU is an agent which has a good possibility of eradicating a diseased erythron but when used alone is not likely to be sufficiently immunosuppressive to permit sustained allogeneic engraftment. In summary, a combination of BU and CY can eradicate the thalassemia and facilitate sustained allogeneic engraftment.

Most patients undergoing transplantation for the treatment of thalassemia have been transfused repeatedly. This lead to sensitization to HLA antigens therefore could increase the risk of rejection as it has been demonstrated in patients with aplastic anemia^18^, ^19^.

The treatment of patients after graft rejection will differ depending on whether the rejection is accompanied by regeneration of host type hematopoiesis or by marrow aplasia.

Preparatory regimens capable of eradicating a diseased marrow and facilitating persistent engraftment are necessarily toxic, and the consequences of successful allogeneic marrow engraftment include acute and chronic graft-vs.-host disease (GVHD), syndromes associated with severe immune incompetence. Regimen-related toxicity has been well described in patients treated by BMT for hematologic malignancies^20^,^21^. The lungs and liver are the organs most at risk for toxicity induced by TBI and BU while the heart is the main site of CY-induced damage. Increasing patient age, previous exposure to cytotoxic agents and the presence of latent viruses such as hepatitis C and cytomegalovirus adversely influence these toxicities. Patients transplanted for the treatment of thalassemia derive benefit from the fact they are usually young and without prior exposure to cytotoxic agents. However, because of previous intensive transfusion therapy they will have a high probability of carrying harmful viruses and have organ damage induced by extreme iron overload.

## Risk Factors and Risk Classes:

Analysis of the influence of pretransplant characteristics on the outcome of transplantation was conducted in 222 patients aged less than 17 years who were all treated with exactly the same regimen^22^, ^23^. In multivariate analysis hepatomegaly more than 2 cm, portal fibrosis and irregular chelation history were associated with a significantly reduced probability of survival. The quality of chelation was characterized as regular when deferoxamine therapy was initiated no later than 18 months after the first transfusion and administered subcutaneously for 8–10 h continuously for at least 5 days each week. Any deviation from this regimen was defined as irregular chelation. On the basis of these risk factors patients were categorized into three risk classes. Class 1 patients had none of these adverse risk factors, class 3 patients had all three and class 2 patients had one or two adverse risk factors.

## The Use of Unrelated Donors:

Results of HCT from unrelated donors for the treatment of malignant disease have improved steadily, mainly due to the introduction of high-resolution molecular techniques for histocompatibility testing and improvements in the management of post-transplant complications. These results have stimulated the use of HCT from unrelated donors for the treatment of hemoglobinopathies. The Italian cooperative group for bone marrow transplantation (Cagliari, Pavia, Pesaro and others) has recently reported results obtained in 32 thalassemic patients (aged 2–28 years) who received bone marrow from unrelated donors selected for similarity with the recipients for extended HLA haplo-types^24^. The probability of thalassemia-free survival was 66% for the entire group and the mortality from causes other than rejection was 25%. Although the difference was not significant, five of the six patients who died belonged to class 3. The incidences of grade 2–4 acute and of chronic GVHD were 41% and 25%, respectively. Patients who shared at least one extended haplotype had less acute or chronic GVHD and better survival.

Recently this group has reported data on 27 adult thalassemia patients (aged 17–37 years) transplanted from unrelated donors selected by high resolution HLA molecular typing^25^. The probability of survival, thalassemia-free survival, transplant-related mortality and rejection were 70 %, 70%, 30% and 4% respectively. The incidence of grade II–IV acute and chronic GVHD was 37% and 27%, respectively. Although limited these data show that unrelated marrow transplatation not only in younger patients but also in adult patients from well-selected donors may offer a success rate similar to those obtained from HLA-identical sibling transplantation.

## Unrelated Cord Blood Transplantation:

Hematopoietic stem cell transplantation from an unrelated cord blood (UCB) donor is, now, standard practice for the treatment of hematological malignancies. The number of UCB transplants has increased dramatically, because several studies have demonstrated that results from HLA-mismatched UCB transplants were comparable to those from HLA-matched unrelated bone marrow transplants in children^26^. Advantages such as faster availability, tolerance of 1–2 HLA mismatches, and low incidence of acute GVHD made UCB transplants attractive for patients with some non malignant diseases^27^. Five patients (aged 2.3– 11.4 years) with β-thalassemia major were given unrelated cord blood transplantation in Taiwan^28^. Cord blood units were mismatched for 2-loci in 2 recipient/donor pairs, and the remaining 3 pairs had a mismatched at 1 locus. Patients were given BU14 mg/kg, CY 200 mg/kg and antithymocyte globulin 30 mg/kg as conditioning and CSP and methylprednisolone as GVHD prophylaxis. Nucleated cell dose ranged from 3.25 to 11.8 x 10^7^/kg. All patients had sustained engraftment with complete donor chimerism. Three patients had grade II-III acute GVHD responsive to steroid treatment. These data are very encouraging and show that patients who need transplantation and do not have a suitable either related or unrelated matched donor can benefit from unrelated cord blood transplatation.

## Pesaro Experience in Patients With β–Thalassemia:

Beetween May 1985 and December 2003, 498 patients <17 years of age had received HLA-identical transplants using regimens containing BU 14mg/kg and CY 200mg/kg. There were 150 class 1 patients, 315 class 2 patients and 33 class 3 patients. The probabilities of survival, thalassemia-free survival, rejection and non-rejection mortality for class 1 patients were 90%, 87%, 3% and 10%, respectively, and for class 2 patients they were 87%, 85%, 3% and 13%, respectively

Between October 1985 and August 2007 five hundred and fifteen class 1 and class 2 patients with median age of 7 years (range 1 to 16 years) were given bone marrow transplantation following conditioning with Bu 3,5 mg/kg/day for 4 consecutive days and CY 50 mg/kg/day for subsequent 4 days. Since 2003 patients aged less than 4 years were also given thiotepa 10 mg/kg/day in addition to BUCY. Four hundred and seventy eight of these patients were given transplant at the Hospital of Pesaro^22^,^29^ and 37 patients received BMT at the University Policlinic of Tor Vergata. Graft- versus-host disease prophylaxis consisted of cyclosporine (CSA) and low-dose methylprednisolone (MP) and since June 2001 CSA, MP and a modified “short course” of methotrexate (MTX).

When class 3 younger patients (age <17 years) treated with the same conditioning as class 1 and class 2 patients (BU14 mg/kg and CY200 mg/kg), showed a low probability of thalassemia- free survival (53%) and a higher probability of non-rejection transplant related mortality (39%) 30. In an attempt to improve results in class 3 patients, new treatment regimens were devised using BU 14mg/kg and lower doses of CY (160 or 120mg/kg). These regimens improved the probability of survival in class 3 younger (age<17 years) patients from 53% to 79%, but were associated with an increase of rejection probability from 7% to 30%, probably due to inadequate immunosuppression and failure to eradicate the massive erythroid hyperplasia characteristic of these patients^29^–^31^.

In April 1997 a new preparative regimen (Protocol 26) was adopted for class 3 patients <17 years of age in an attempt to decrease the higher rejection rate through reducing erythroid expansion and increasing immunosuppression over time to avoid unacceptable drug toxicity during conditioning regimen^32^. Protocol 26 was devised on the assumption that preparation with BU 14mg/kg and CY 160mg/kg was inadequate to eradicate thalassemic hematopoiesis in class 3 patients <17 years of age. Patients were given azathioprine 3mg/kg and hydroxyurea 30mg/kg daily from day -45 from the transplant, fludarabine 20mg/m2 from day -17 through day-13, followed by the administration of BU 14mg/kg total dose and CY 160mg/kg total dose. Graf-tversus-host disease prophylaxis consisted of CSA, low-dose methylprednisolone and a modified “short course” of MTX. As reported in Table 1, the probabilities of survival, thalassemia-free survival, rejection and non-rejection mortality in 33 such patients treated with Protocol 26 were 93%, 85%, 8% and 6%, respectively 32.

Adult thalassemia patients have more advanced disease with both disease and treatment related organ complications mainly due to prolonged exposure to iron overload. From November 1988 through September 1996, 107 patients older than 16 years received transplants from matched donors. The median age for this population was 20 years (range 17–35 years). The probability of survival, thalassemia-free survival, rejection and non-rejection mortality for the entire group of these patients were 66%, 62%, 4% and 37% respectively^33^, ^34^.

From April 1997 all adult patients were prepared for transplantation according to Protocol 26 with the only difference that the dose of cyclophosphamide was reduced to 90 mg/kg total dose. The probability of survival, thalassemia-free survival, rejection and non-rejection mortality in 15 high risk group patients were 65%, 65%, 7% and 28% respectively^35^.

## The Experience of the International Center for Transplantation in Thalassemia and Sickle Cella Anemia in Roma:

This experience is characterized by two peculiarities: patients were ethnically very heterogeneous and the vast majority of these patients were not regularly transfused/chelated and therefore were highly sensitized due to RBC transfusions without leukodepletion filters.

Therefore these patients could have a higher risk of graft rejection as a result of sensitization to HLA antigens. From June 2004 through September 2009, one hundred and forty five consecutively patients (84 male and 61 female) with thalassemia and sickle cell anemia were given BMT at the Roma Center. Table 1 shows the geographic distribution of the patients.

## RESULTS

### HLA Identical Patients:

Class 1 and class 2 patients under 4 years of age were given BU14CY200 and thiotepa 10 mg/kg total dose, while patients ≥ 4 years of age received BU14CY200 as preparatory regimen. Prophylaxis against GVHD consisted of CSP, low dose of methylprednisolone and a short course of MTX. Class 3 patients were prepared for BMT according to Protocol 26. Since June 2006, all patients were given targeted dose of intravenous Busilvex instead of oral Busulfan. The treatment protocols in use are shown in Table 2.

The preliminary data relative to 106 HLA identical patients shows that the probabilities of survival, thalassemia-free survival, rejection and non-rejection mortality in class 1 patients (20 patients) were 95%, 90%, 5% and 5% respectively. 38 patients in class 2 showed a survival, thalassemia-free survival, rejection and non-rejection mortality of 89%, 84%, 12% and 5% respectively. In 48 class 3 patients we observed a survival, thalassemia-free survival, rejection and non-rejection mortality of 87%, 81% 11% and 9% The Rome experience in HLA identical patients confirmed the results obtained in Pesaro.

### Second Transplant in Patients With Thalassemia Recurrence:

There is a substantial incidence of graft failure in patients with thalassemia after myeloablative conditioning regimens especially in class 3 patients in whom its incidence could be as high as 8–38.5%. Most patients with graft failure have recurrence of thalassemic marrow.

We have recently published data on 16 patients with thalassemia recurrence following rejection of the first graft who were given second transplants. The median of age was 9 years (range, 4–20). These patients were given second transplant using BM (n=7) or PBSC (n=9) after preparation with a new treatment protocol. All but two patients received stem cells from the same donor. The median interval between two transplants was 28 months (range, 8–204). The sustained engraftment rate was high (94%) with only one patient having primary graft failure. The probability of overall survival, event-free survival, TRM and graft failure were 79, 79, 16 and 6%, respectively. There were three transplant-related deaths. This intensified treatment protocol was well tolerated with no significant increase in toxicity^36^.

### Haploidentical Hematopoietic Stem Cell Transplantation from a Mismatched Family Member in Patient With β-Thalassemia:

Approximately 25% to 30% of patients with thalassemia could have an HLA matched related donor. As HSCT is the only cure for thalassemia there is need to develop alternative stem cells donations. Our past experience with BMT from alternative donors for 29 patients with β-thalassemia major who received phenotypically matched grafts or haploidentical grafts mismatched for one, two, or three antigens was characterized by higher graft failure (55%), and low thalassemia-free survival (21%)^37^. Haploidentical hematopoietic stem cell transplantation from a mismatched family member (HaploHSCT) donor offers an alternative option for patients who lack an human leukocyte antigen (HLA)-matched donor. The main obstacles are graft rejection, delayed immune reconstitution, graft-versus-host disease (GvHD) and higher frequency of opportunistic infections. Assuming that the immunotollerance established during the pregnancy might help to bypass the HLA disparity we started a transplant program of haploidentical T-cell depleted HCT from mother to child for thalassemia in 2002. Feto-maternal microchimerism suggests immunological tolerance between mother and fetus. Thus, we performed primary hematopoietic stem-cell transplantation (HSCT) from mismatched mother to thalassemic patient without an HLA-identical donor.

We have recently published data relative to 22 patients with thalassemia major^38^.

These patients were conditioned with 60 mg/kg hydroxyurea and 3 mg/kg azathioprine from day -59 to -11, 30 mg/m(2) fludarabine from day -17 to -11, 14 mg/kg busulfan starting on day -10, and 200 mg/kg cyclophosphamide, 10 mg/kg Thiotepa, and anti-thymocyte globulin daily from day -5 to -2. 14 patients received CD34(+) mobilized peripheral and bone marrow progenitor cells; eight patients received marrow graft selected PBSC CD34(+) and BM CD3/CD19 depleted. T-cell dose was adjusted to 2 x 10(5)/kg by fresh marrow cell add-back at the time of transplant. Both groups received cyclosporine for graft versus host disease (GVHD) prophylaxis for two months post transplant. Two patients died (cerebral EBV lymphoma or CMV pneumonia), six patients reject their grafts, and 14 showed full chimerism with functioning grafts at a median follow-up of 40 months. None of the 14 patients who showed full chimerism developed acute or chronic GVHD. In a part of these patients we performed an immunological study to investigate immunological tolerance and immune reconstitution post transplantation.

In haploidentical hematopoietic transplantation, donor-versus-recipient NK cell alloreactivity derives from a mismatch between donor NK clones bearing inhibitory Killer Cell Ig-like Receptors (KIRs) for self HLA class I molecules and their HLA class I ligands (KIR ligands) on recipient cells. The mechanism whereby alloreactive NK cells exert their benefits in transplantation has been elucidated. The infusion of alloreactive NK cells 1) ablates recipient T cells which reject the graft, and 2) ablates recipient dendritic cells (DCs) which trigger GvHD, thus protecting from GvHD. NK cell alloreactivity also boosts very rapid rebuilding of donor adaptive immunity to infections. Immuno haematological reconstitution was therefore analysed in some patients after HLA-haploidentical transplant^39^. T and B cell depletion was carried out with CD34+ coated magnetic microbeads and the CliniMACS device (Miltenyi Biotec©) from peripheral blood and bone marrow of donors (the mothers) and resulted in grafts consisting of stem cells and effector cells (NK cells, monocytes) with the addition of bone marrow mononuclear cells (BMMNCs 3 x 10^5^/kg of the recipient). A total of 12 pediatric patients with β-thalassemia received T and B cell depleted transplants from their haploidentical mothers with a median number of 15 x 10^6^ CD34 stem cells. To analyse the mechanisms involved in immunological reconstitution post transplant, we analysed T cell subsets by flow cytometry, particularly NK sets (CD3-CD56+, CD3-CD16+ and CD56+CD16+ NK cells) at day + 20 and + 60 post transplant. Day + 20 post transplant, the patients had significantly lower CD4+ T cells in comparison to the controls, whereas CD8+ T cells numbers did not statistically differ between patients and controls. NK cells were among the first lymphocytes to repopulate the peripheral blood, and up to 70% of these cells were CD3-CD56+^bright^ cells. Interestingly, a direct correlation has been observed between the percentages of CD56+CD16+ NK subset and the BM engraftment and in all the patients the origin of the NK subsets was from the mothers. Day + 60 post transplant an increase in the percentages of CD4+ T cells, naïve CD4+ cells and in thymic naïve Th cells were observed [Isgrò A. et al submitted]

### The Sickle Cell Anemia (SCA):

The SCA is one of the most common autosomal recessive disorders in the world. A mutation in the β–globin gene substitutes valine for glutamic acid at position 6 in the β–globin chain of haemoglobin A, resulting in a hemoglobin called S. Approximately 8 % of black Americans are heterozygous for β-globin gene mutated and has sickle cells trait. 1 in 600 is homozygous and has sickle cell disease. 40 In sub –Saharn Africa an estimated 40 to 60 % of population is heterozygous and 1 to 4 child have the disease 41. However this can become a world-wide social and healthy problem because the migration from Africa is increased during the last years. Moreover these patients can have many acute events as vaso-occlusive pain crisis, acute chest syndrome, caused by physical and adhesive entrapment of red cells containing Hemoglobin S in the microcirculation. With increasing age renal failure, stroke, avascular necrosis of bone, and pulmonary hypertension begin to appear. SCD is an increasing healthy problem in the European countries.

### Bone Marrow Transplantation for Sickle Cell Disease:

Currently, stem cell transplantation (SCT; from bone marrow, peripheral blood and umbilical cord blood) is the only treatment that offers an effective cure for SCD patients. Although the first BMT for SCD was carried out in 1984, currently approximately 250 affected patients received transplant, a far lower number than undertaken during the same period for thalassemia. There are some barriers to widely applying SCT for SCD patients. The absence of HLA -identical sibling donor is the major obstacle for expanding this curative treatment to many potential candidates with this disease. In fact, in one study, only 14% of eligible patients, who had siblings HLA typed, had an HLA-identical donor. For this reason, alternative sources of hematopoietic stem cells, such as unrelated donor marrow or umbilical cord blood, are needed. The higher individual variability of disease severity, and some progress of pharmacological therapy such as hydroxiurea treatment are also reasons for the delay or refuse of SCT by physicians and their patients. Another important obstacle is that there are no universally accepted criteria to identify the subset of patients at higher risk for early death. This has led several groups to offer SCT to patients with a significant clinical complication of SCD (stroke or recurrent painful episodes). In all these studies main indications for transplant were vasoocclusive complications such as stroke, recurrent episodes of acute chest syndrome and/or painful crisis.

### Rome Results in Sickle Cell Disease:

Since 2004 in we have performed BMT on 7 patients with SCD in our center. Patients (median age of 13 years; range 2–16 years) received BU/CY/ATG as conditioning regimen and CSA+short MTX as GvHD prophylaxis. All patients had a sustained engraftment. Six patients are alive and well while 1 patient died at 1 year after BMT at home. (Table 3)

### Conclusions:

Hematopoietic cell transplantation is the only treatment able to definitively cure thalassemia and sickle cell disease. Class 1 patients with thalassemia have a very high probability of cure with a very low early and late morbidity and mortality. Therefore there is no reason to deny these patients the advantages of a life free from daily tedious, expensive and uncomfortable therapy. We still do not know the probability that a patient receiving conventional therapy will deteriorate into a worse risk category, but the fact is that transplant centers are often confronted with patients in risk classes 2 and 3 who represent failures of conventional treatment. Delaying transplantation until the patient is in a risk category beyond class 1 substantially reduces the probability of transplant success and jeopardizes the reversibility of liver and cardiac damage. We therefore believe that all patients with β-thalassemia who have HLA-identical related donors should be transplanted as soon as possible.

Patients without such donors who have well-selected unrelated donors should also be considered for HCT. Our recent results suggest that maternal haploidentical HSCT is feasible for patients with thalassemia who lack a matched related and unrelated donor. The Rome experience confirmed the results obtained in Pesaro, and most importantly, showed the reproducibility of the Pesaro experience in other centers and with the patients ethnically heterogeneous.

## Figures and Tables

**Table 1.** Country of origin of 145 patients transplanted at Roma Center from 2004 through 2009.

**Country of origin**	**N° of patients**

AFGHANISTAN	2
ALBANIA	3
ARGENTINA	4
BAHREIN	3
BULGARIA	1
CANADA	1
CYPRUS	2
EGYPT	10
IRAQ	32
ITALIA	2
KUWAIT	31
LEBANON	25
MADAGASCAR	1
MALDIVES	10
NEPAL	1
PAKISTAN	3
PALESTINE	7
QATAR	1
ROMANIA	2
RUSSIA	1
SYRIA	2
USA	1
TOTAL	145

**Table 2.** Current treatment protocols.

**Risk Classes**	**Pre Conditioning**	**Conditioning**	**GvHD prophylaxis**
Class 1 & Class 2 > 4 yrs	none	Pc 6 *Bu14 CY200	CSA/MP/sMTX
Class 1 & Class 2 ≤ 4 yrs	none	Pc 6.1 Bu16 TT10CY200	CSA/MP/sMTX
Class 3 <17 yrs before February 2007	d-35 -11: AZ,HU d-16 -11: Flu 100 G-CSF, DFO	Pc 26 Bu14 CY160	CSA/CY/MP/sMTX
Class 3 <17 yrs Since February 2007	d-35 -11: AZ,HU d-16 -11: Flu 150 G-CSF, DFO	Pc 26 modified i.v.Bu TT10CY160	CSA/CY/MP/sMTX
Adults (age > 17 yrs) Class 1-3. since 2/2007	d-35 -11: AZ,HU d-16 -11: Flu 150 G-CSF, DFO	Pc 26 modified i.v.Bu TT10CY90	CSA/CY/MP/sMTX

Bu=Busulfan -

* Since June 2006 all patients receive i.v. Busulfan (Busilvex); CY =Cyclophosphamide; MP= methylprednisolone; CSA=Cyclosporine; MTX= methotrexate; TT=thiotepa; Flu=Fludarbine; AZ=Azathioprine; Hu=hydroxyurea; DFO=deferoxamine, G-CSF=granulocyte colony-stimulating factor

**Table 3.** BMT on 7 patients with SCA transplanted at the Roma Center.

	**Age, yrs**	**F-up, mo**	**Outcome**

**1**	13	24	alive & cured
**2**	13	16	alive & cured
**3**	14	12	alive & cured
**4**	10	10	alive & cured
**5**	16	12	Died at home
**6**	2	6	alive & cured
**7**	12	1,5	alive &cured
